# IgA Nephropathy: Mechanisms, Risk Stratification, and Precision Therapy

**DOI:** 10.3390/diagnostics16091259

**Published:** 2026-04-22

**Authors:** Sami Alobaidi

**Affiliations:** Department of Internal Medicine, University of Jeddah, Jeddah 21493, Saudi Arabia; salobaidi@uj.edu.sa

**Keywords:** IgA nephropathy, oxford classification, risk stratification, biomarkers, proteinuria, supportive care, complement inhibition, clinical trials

## Abstract

IgA nephropathy is the most common primary glomerulonephritis worldwide and a leading cause of chronic kidney disease and kidney failure, with geographic and ancestral variation and a course ranging from asymptomatic urinary abnormalities to progressive loss of kidney function. This narrative review links the multi-hit model to risk stratification, biomarkers, current management, and emerging therapies, and highlights implementation gaps. Risk assessment is longitudinal, prioritizing proteinuria and estimated glomerular filtration rate trajectories and integrating Oxford MEST-C, prediction tools, and biomarker and multi-omics approaches, while recognizing limitations in histologic reproducibility and model calibration. Current management is anchored in optimized supportive care aimed at sustained proteinuria reduction and kidney protection, including intensive blood pressure control with maximal tolerated renin–angiotensin system blockade, dietary sodium restriction and lifestyle measures, and sodium–glucose co-transporter 2 inhibitors for eligible patients. For selected higher-risk patients with persistent proteinuria despite optimized supportive care, immunomodulatory strategies are discussed, including systemic corticosteroids and targeted-release budesonide (Nefecon), emphasizing structured toxicity risk mitigation and cautioning against assuming interchangeability among alternative oral budesonide formulations. Emerging therapies are organized around mechanism-aligned targets across the BAFF/APRIL axis, complement pathways, and endothelin-based approaches, with growing interest in sequencing and combination regimens layered on supportive care. Key gaps include reliance on surrogate endpoints, limited long-term durability and safety data, and uneven evidence for special populations.

## 1. Introduction

IgA nephropathy (IgAN), first described by Berger and Hinglais in 1968, is one of the most common primary glomerulonephritides worldwide and a leading cause of chronic kidney disease (CKD) and kidney failure, with a high burden in East Asia and substantial prevalence in Europe [1,2]. Although once considered indolent, historical cohorts show that up to 40% of patients reach kidney failure over two to three decades [1,2,3,4,5]. This marked regional and ancestral variation—driven by differences in genetic susceptibility, environmental exposures, and access to biopsy and screening—underscores the complex interplay between inherited risk and mucosal immune dysregulation [1,2,4,5].

The pathogenesis of IgAN involves a multi-hit autoimmune process driven by the production of galactose-deficient IgA1 (Gd-IgA1) and ensuing immune complex formation. Immune complex deposition triggers complement-mediated inflammation, ultimately leading to glomerular injury and fibrosis [3,6,7].

Therapeutic advances have shifted management from nonspecific immunosuppression toward pathway-directed approaches. These include targeted-release budesonide (TRB; evaluated in NefIgArd), which is intended to modulate distal ileal mucosal immune activity and thereby may modulate pathogenic IgA pathways [8]; endothelin receptor antagonists (ERAs), including sparsentan (PROTECT) [9] and atrasentan (ALIGN) [10], as adjuncts to optimized supportive care; B-cell modulation via BAFF/APRIL inhibition with atacicept [11]; and selective complement inhibition at proximal (factor B, iptacopan), terminal (C5, cemdisiran), and lectin pathway targets (mannan-binding lectin-associated serine protease-2) [5,7,12].

Contemporary trials typically use early proteinuria reduction as a surrogate endpoint, complemented by longer-term estimated glomerular filtration rate (eGFR) slope. This strategy requires optimized background supportive care, aligning trial design with disease biology and the stricter therapeutic targets emphasized in the 2025 KDIGO guidelines [5,10,12,13].

Collectively, these epidemiologic patterns, mechanistic insights, and therapeutic advances motivate a contemporary, globally informed review of IgAN that integrates updated insights in epidemiology, pathogenesis, risk stratification, and the evolving treatment landscape [2,4].

This narrative review was informed by a targeted search of PubMed/MEDLINE, Scopus, Web of Science, and Google Scholar using combinations of terms related to IgA nephropathy, pathogenesis, biomarkers, risk stratification, and treatment, primarily focusing on the literature published in recent years while including seminal earlier references when directly relevant.

## 2. Epidemiology and Global Burden

**Figure 1:**
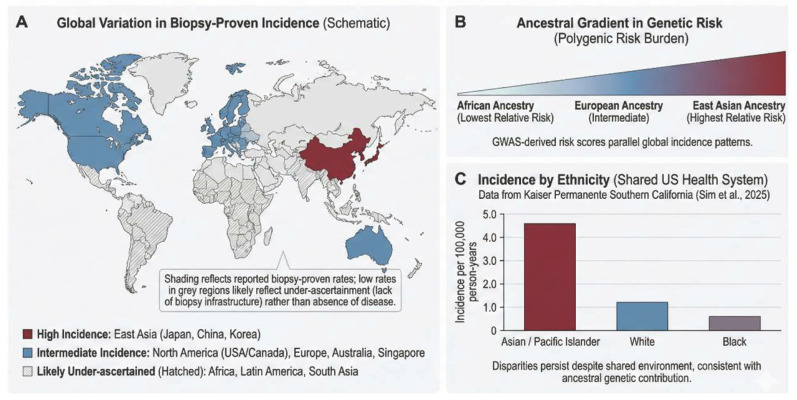
Global variation in biopsy-confirmed IgAN incidence and ancestry-related genetic susceptibility. (**A**) Schematic regional distribution of reported biopsy-proven IgAN incidence. (**B**) Conceptual gradient of genome-wide association study (GWAS)-derived polygenic risk across ancestries. (**C**) IgAN incidence by ethnicity within a single U.S. health system (Kaiser Permanente Southern California) [20].

Incidence varies markedly across regions and ancestries, but the frequency and threshold for kidney biopsy are major determinants of the reported incidence and prevalence of IgAN in any given population. Differences in biopsy practice, healthcare access, registry capture, and diagnostic infrastructure therefore strongly influence epidemiologic estimates, alongside genetic and environmental risk [1,14,15]. Population-based incidence estimates range from 0.06 per 100,000 person-years in South Africa to approximately 10.5 per 100,000 person-years in Australia [1,14]. In Denmark, a population-based cohort of 1298 biopsy-verified adult IgAN cases (2002–2023) reported a threefold rise in incidence, with prevalence reaching 261 per 1,000,000 in 2023, without clear improvement over time in 5-year KFRT or mortality [16]. Regional incidence patterns, together with evidence that ancestral genetic susceptibility contributes to geographic and ethnic variation within shared health systems, are summarized in Figure 1. These observations underscore the need for harmonized surveillance and equitable diagnostic tools globally.

East Asia consistently reports the highest biopsy-proven incidence, driven partly by national urine screening programs and lower thresholds for renal biopsy [14]. Recent biopsy registries from China reveal that IgAN accounts for nearly 40% of primary glomerulonephritis diagnosed on kidney biopsy, remaining the dominant glomerulonephritis in the region despite a slight decrease in weighted prevalence after 2010 [1,17]. In contrast, sub-Saharan Africa and parts of Latin America report low biopsy-proven incidence, likely reflecting limited diagnostic infrastructure and under-diagnosis rather than true absence of disease [2,14].

In the Middle East, fragmented registries and limited biopsy utilization likely underestimate the true burden [2,18]. Within shared health systems, ethnic disparities persist: within an integrated United States health system (Kaiser Permanente Southern California, Pasadena, CA, USA), biopsy-confirmed IgAN was more common among Asian/Pacific Islander than White or Black individuals [15,19,20]. These differences likely reflect combined effects of genetic susceptibility, environmental exposures, and system-level factors [14].

Despite growing recognition of IgAN’s global reach, many regions still lack reliable epidemiologic data due to absent national registries and inconsistent case definitions. A recent multinational patient-reported outcomes study (N = 1515) highlighted IgAN’s broad geographic distribution and underscored persistent gaps in early diagnosis and long-term monitoring across major global economies [2,21]. While tools such as the International IgAN Prediction Tool (IIgAN-PT) help to standardize risk assessment, they are not substitutes for harmonized global surveillance systems, which remain a critical priority to guide equitable policy and research investment [2].

## 3. Pathophysiology: The Multi-Hit Model

**Figure 2:**
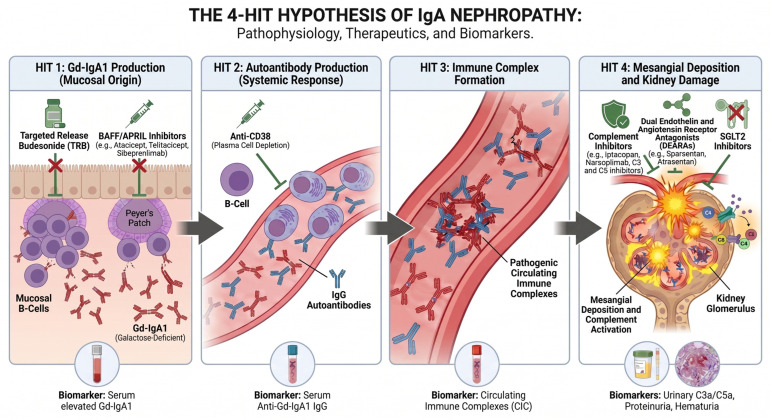
Multi-hit pathogenesis of IgAN with stage-aligned therapeutic targets and representative biomarkers. Schematic overview of Hits 1–4 with representative biomarkers and major therapeutic target classes aligned to each step; therapy labels denote target classes rather than exhaustive drug lists.

**Figure 3:**
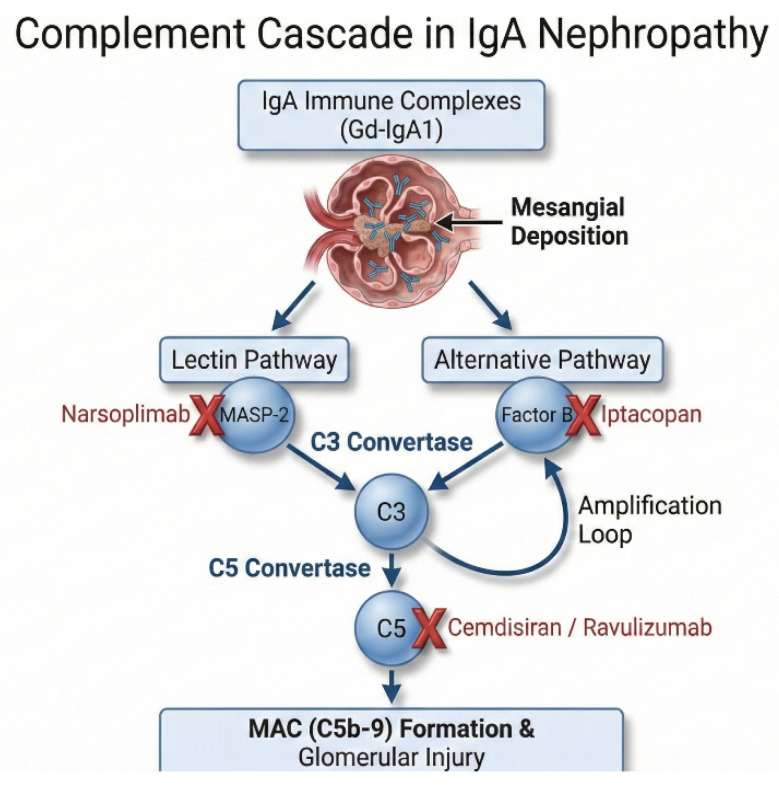
Complement activation and therapeutic targets in IgAN. The schematic depicts lectin- and alternative-pathway activation, convergence at C3/C5 convertases with an amplification loop, and downstream MAC (C5b-9) formation. Target nodes and example agents are shown (MASP-2/narsoplimab, Factor B/iptacopan, and C5-directed approaches including ravulizumab and cemdisiran).

The pathogenesis of IgAN is best conceptualized by a four-hit, multi-step hypothesis that integrates genetic predisposition, environmental triggers (including gut microbiota dysbiosis) [22], and mucosal immune dysregulation to explain the formation of nephritogenic immune complexes and subsequent glomerular injury [23,24,25,26]. To link this framework to actionable biomarkers and therapeutic leverage points, Figure 2 summarizes the multi-hit cascade and highlights stage-aligned targets. In this framework, complement activation is positioned as an effector mechanism within Hit 4 and is expanded in Figure 3.

### 3.1. First Hit: Aberrant Glycosylation of IgA1

The initial event in IgAN pathogenesis is the overproduction of Gd-IgA1, characterized by under-galactosylated hinge-region O-linked glycans that expose N-acetylgalactosamine residues and confer neoantigenic potential [23,24,25]. Circulating and urinary Gd-IgA1 are consistently elevated in IgAN and discriminate from healthy controls in meta-analyses, while familial aggregation supports a heritable glycosylation defect [26,27]. Glycomic profiling further identifies site-specific O-glycan changes linked to renal function, supporting Gd-IgA1 as a mechanistic hallmark and measurable biomarker [25].

Mechanistic studies implicate B cells as a systemic source of Gd-IgA1. Epstein–Barr virus (EBV)-immortalized IgA1-secreting cells from IgAN patients produce IgA1 with defective galactosylation and reduced glycosyltransferase activity [24]. Inflammatory signaling may also contribute to this enzymatic defect, as interleukin-6 (IL-6)–Janus kinase 2 (JAK2)/signal transducer and activator of transcription 3 (STAT3) activation has been linked to suppression of C1GalT1/Cosmc and increased Gd-IgA1 secretion in IgA^+^ B cells models, alongside evidence of IL-6 pathway activation in patient-derived immune cells [28]. Genetic susceptibility likely modulates this phenotype, with immune-regulatory loci such as human leukocyte antigen (HLA)/major histocompatibility complex (MHC) showing strong association signals in genome-wide studies [29]. Together, this first hit generates the biochemical substrate that promotes downstream anti-glycan immune recognition and immune complex formation [23,24,25,26,29].

### 3.2. Second Hit: Autoantibody Formation

The second stage in IgAN pathogenesis is a break in immune tolerance that produces anti-Gd-IgA1 IgG/IgA autoantibodies that bind hinge-region glycans to form pathogenic circulating immune complexes (CICs) [30,31,32,33]. This step is crucial for the development of clinically recognizable IgAN, as elevated Gd-IgA1 alone appears insufficient to cause overt disease. Compared with healthy controls and unaffected relatives with elevated Gd-IgA1, the anti-Gd-IgA1 autoantibody signal supports dysregulated immunity rather than antigen excess as the driver of disease [3,27]. Thus, the second hit represents the critical transition from aberrant IgA1 production to the formation of nephritogenic immune complexes capable of initiating glomerular injury. Anti-Gd-IgA1 antibodies are affinity-matured and autoreactive, consistent with failed B-cell tolerance checkpoints [31,32,33]. TRDMT1-mediated 5-methylcytosine (m5C) modification has been implicated in IgA class switching, and pathway inhibition reduces IgA production and attenuates IgAN-like disease in vivo [34]. Elevated BAFF and APRIL sustain plasma cell differentiation and survival, supporting ongoing autoantibody production and CIC formation [35,36]. Collectively, this second hit links aberrant IgA1 production to immune complex-primed mesangial deposition and glomerular injury.

### 3.3. Third Hit: Immune Complex Deposition and Mesangial Activation

Immune complexes containing Gd-IgA1 and anti-glycan autoantibodies deposit within the mesangium, where overexpressed CD71 (transferrin receptor) and transglutaminase-2 (TG2) mediate high-affinity binding/retention and initiate injury [37,38]. Soluble CD89 (sCD89)–IgA1 complexes further amplify this CD71/TG2 axis, despite the absence of membrane CD89 on mesangial cells [38]. Downstream mitogen-activated protein kinase/extracellular signal-regulated kinase (MAPK/ERK) and nuclear factor κB (NF-κB) activation promotes mesangial proliferation, IL-6 and monocyte chemoattractant protein-1 (MCP-1) release, and extracellular matrix deposition (collagens, fibronectin), linking immune complex load to sclerosis [6]. Patient-derived IgA1 immune complexes reproduce these effects in human mesangial cells and ex vivo models, increasing IL-6 and transforming growth factor-β (TGF-β) and priming complement engagement [6].

### 3.4. Fourth Hit: Effector Mechanisms and Complement Activation

Complement activation is a major effector axis linking immune complex deposition to glomerular injury (Figure 3). Evidence includes glomerular deposition of C3, properdin, and C5b-9 (membrane attack complex; MAC) and urinary soluble C5b-9 (sC5b-9) levels that correlate with disease severity [39,40]. As summarized in Figure 3, mesangial IgA immune complexes engage the lectin and alternative pathways and converge on C3/C5 activation to generate MAC.

Therapeutically, Phase 3 proteinuria reduction with Factor B inhibition (iptacopan) supports the alternative pathway as a clinically tractable target, whereas MASP-2 inhibition (narsoplimab) failed in Phase 3, suggesting redundancy or distinct roles in human disease [12,41]. Accordingly, current strategies emphasize the most consistently supported nodes, including proximal alternative pathway inhibition (Factor B) and terminal complement blockade (C5) [12,42].

The major complement activation steps and corresponding therapeutic targets are summarized in Figure 3.

### 3.5. Mucosal–Microbiome Axis in IgAN

Aberrant mucosal immunity is central to IgAN. Antigenic stimulation within gut-associated lymphoid tissue (GALT) and the tonsillar compartment drives class switching and Gd-IgA1 overproduction via toll-like receptor 9 (TLR9) and BAFF/APRIL signaling, and patient-derived IgA1-secreting cells produce polymeric IgA1 with abnormal O-glycans, consistent with mucosal plasma-cell dysregulation of C1GalT1/Cosmc [3,6,24,43]. Supporting a tonsil-centered mucosal immune contribution, tonsillar T-cell receptor repertoire features in IgAN have been linked to tonsillar Gd-IgA1 levels and IgA–microbiome interactions [44]. Mucosal imprinting and trafficking through integrin α4β7 and chemokine receptor 9 (CCR9) promote dissemination of Gd-IgA1-secreting B cells from gut/tonsil niches, while IgAN-associated dysbiosis—reduced diversity and depletion of short-chain fatty acid-producing taxa—tracks with clinical severity and therapeutic response [45,46,47,48]. Recent mechanistic data further suggest that mucin-degrading gut bacteria may contribute to post-translational modification of IgA1, generating deglycosylated autoantigenic epitopes that may promote autoreactive antibody responses and further support the gut–kidney axis hypothesis in IgAN [49]. Multi-omics profiling of ileocecal and circulating immune cells localized Gd-IgA1-enriched IgA^+^ B-cell responses to the terminal ileum and identified increased circulating IgA^+^ integrin β7^+^ plasmablasts/plasma cells correlating with disease severity [50]. Mucosal inflammatory signaling may further amplify these programs. IL-6–JAK2/STAT3 activation has been linked to suppression of C1GalT1/Cosmc and increased Gd-IgA1 secretion in IgA^+^ B-cell models, with supportive evidence of IL-6 pathway activation in patient-derived immune cells [28].

Barrier dysfunction may increase translocation of microbial products such as lipopolysaccharide (LPS), and toll-like receptor (TLR) signaling associates with APRIL/BAFF up-regulation, reinforcing aberrant IgA class switching [48,51]. Recent (2025) bidirectional Mendelian randomization analyses support a causal link from specific gut microbiota to IgAN, identifying taxa such as Barnesiella as risk factors while others (Alistipes) appear protective [52]. Downstream, engagement of FcαRI (CD89) by Gd-IgA1 induces receptor shedding and formation of sCD89–IgA complexes, which circulate and deposit in the mesangium [53,54]. Together, these pathways support mucosa-targeted approaches (e.g., targeted-release budesonide) and a rationale for adjunctive microbiome modulation, including probiotics or dietary interventions [46,55]. Consistent with this rationale, oral fecal microbiota transplantation has been explored in IgAN in an exploratory clinical trial with short-term reduction in proteinuria, but the evidence remains preliminary and investigational [56].

### 3.6. Genetic Risk Loci and Polygenic Predisposition

IgAN is a polygenic disorder in which susceptibility loci converge on mucosal immune, complement regulatory, and host defense pathways. Early GWAS delineated the initial genetic architecture, identifying loci including the HLA region (e.g., HLA-DQA1/DQB1), CFHR3/CFHR1, CARD9, ITGAM–ITGAX, VAV3, and DEFA [35]. These studies also revealed ancestry gradients, with higher frequencies of risk alleles in East Asians and protective alleles at loci such as HORMAD2 being consistent with lower prevalence in African populations [35].

A large-scale trans-ethnic GWAS later expanded this landscape, identifying 16 novel loci including REL, CD28, PF4V1, and TNFRSF13B and further implicating NF-κB signaling and cytokine regulation in pathogenesis [57]. Genetic analyses have also refined the “first hit” by mapping loci that regulate serum Gd-IgA1 levels (e.g., C1GALT1), supporting a direct genetic contribution to the glycosylation defect independent of general immune activation [2].

For risk prediction, polygenic risk scores (PRS) derived from these loci are evolving from research tools to potential clinical adjuncts. While early scores only modestly stratified susceptibility, newer models integrating expanded single-nucleotide polymorphism (SNP) panels (~21–55 SNPs) show stronger correlations with clinical severity (earlier age of onset, degree of hematuria) and histological activity (mesangial hypercellularity), suggesting a future role in stratifying progression risk alongside conventional biomarkers [2,58]. Ongoing expansion of genomic datasets to include diverse populations remains critical for refining PRS performance and supporting equitable precision medicine.

### 3.7. Translational Implications: Linking Pathogenesis to Therapeutic Targets

Advances in understanding the multi-hit pathogenesis of IgAN increasingly map each “hit” to therapeutic entry points, supporting a shift from nonspecific immunosuppression toward pathway-directed strategies [3,43]. Upstream approaches aim to reduce mucosal Gd-IgA1 production and the generation of anti-glycan autoantibodies, whereas downstream strategies target immune complex handling and complement activation to limit inflammatory and fibrotic remodeling [53,54]. The persistence and recurrence of IgAN after kidney transplantation highlight durable mucosal immune memory, strengthening the rationale for gut-directed and microbiome-modulating interventions [3,8,43]. Finally, integrating biomarkers (e.g., serum Gd-IgA1, anti-Gd-IgA1 autoantibodies, and sCD89) into clinical trials can enable patient stratification and response monitoring, accelerating individualized, mechanism-based therapy [3,54,59]. These pathogenesis-to-therapy links also inform clinical phenotypes and frame the diagnostic and risk-assessment approach discussed below.

## 4. Clinical Presentation and Diagnosis

### 4.1. Clinical Presentation

IgAN presents across a broad spectrum, ranging from asymptomatic urinary abnormalities to rapidly progressive glomerulonephritis (RPGN). The most frequent initial manifestation—particularly in children and young adults—is macroscopic hematuria occurring synpharyngitically (during or shortly after upper respiratory or gastrointestinal infections); acute kidney injury (AKI) during these episodes is often driven by tubular red blood cell obstruction and may be reversible with supportive care, in contrast to the rarer AKI-RPGN phenotype driven by crescentic glomerular injury [60].

Many patients present with persistent microscopic hematuria with or without sub-nephrotic proteinuria. Nephrotic syndrome is uncommon (~5% of cases) and usually reflects either a “minimal change disease-like” podocytopathy or secondary focal segmental glomerulosclerosis (FSGS) due to chronic scarring [61]. Hypertension is frequent at diagnosis, often unmasking otherwise silent chronic disease, and rarely may be severe or malignant.

Presentation varies by age and region: children typically show episodic gross hematuria with preserved renal function; adults more often exhibit persistent microscopic hematuria and proteinuria with variable renal impairment; and older adults frequently present with advanced CKD and hypertension [60]. In East Asia (e.g., Japan), school-based urine screening enables early detection of asymptomatic microhematuria/mild proteinuria, whereas Western centers typically biopsy only when proteinuria exceeds 0.5 g/day or eGFR declines—thereby enriching Western cohorts for more advanced disease [4,62].

Prognostic weight differs between hematuria and proteinuria. Persistent proteinuria (>0.5–1.0 g/day) and elevated blood pressure are the strongest predictors of renal decline and the primary indications for kidney biopsy and treatment [62,63]. Conversely, isolated microscopic hematuria (without proteinuria or eGFR decline) is generally considered a low-risk phenotype and is not a guideline indication for biopsy, though long-term surveillance is warranted as a subset may progress [61,64].

### 4.2. Diagnostic Evaluation

Diagnosis requires kidney biopsy demonstrating dominant or co-dominant mesangial IgA immune complex deposition, interpreted in a clinicopathologic context to exclude secondary causes. Typical findings include mesangial IgA with frequent C3 and variable IgG/IgM on immunofluorescence, mesangial hypercellularity on light microscopy, and mesangial electron-dense deposits on electron microscopy [60,65]. Immunohistochemical staining for Gd-IgA1 (KM55) may serve as a biopsy-based adjunct in selected cases and can support the differential diagnosis of IgA-containing glomerular diseases. However, its specificity is imperfect, and it should not be used in isolation to distinguish primary IgAN from secondary or infection-related mimics [66,67,68].

Because mesangial IgA dominance is not pathognomonic, clinicopathologic integration should guide diagnosis, after which the Oxford MEST-C score can be applied in confirmed IgAN [65]. AI-enabled deep learning approaches have been explored as adjunctive tools to distinguish IgAN from diabetic nephropathy on renal pathology images, but these methods remain investigational and are not currently used in routine clinical diagnosis [69]. Ultrasound shear wave elastography (SWE) has also been evaluated as a non-invasive adjunct; however, its role remains exploratory and requires broader external validation before clinical implementation [70].

Biopsy timing differs by age and health system, shaping the apparent severity at diagnosis [60]. Although KDIGO 2025 states that no biomarkers are validated for routine clinical diagnosis, candidates such as urinary TYROBP/HCK and serum or urinary Gd-IgA1 have shown encouraging diagnostic performance in exploratory or validation cohorts, supporting continued investigation into future non-invasive diagnostic strategies rather than current routine clinical use [26,62,71].

### 4.3. Oxford Classification and Histopathologic Risk Stratification

The Oxford Classification (MEST-C) standardizes histopathologic reporting in IgAN and has been refined and validated, with prognostic value but recognized reproducibility limits for some lesions—particularly endocapillary hypercellularity—across diverse populations [63,65,72,73,74].

MEST-C should be applied only after excluding secondary causes and integrating clinical context; in practice, it complements rather than replaces multivariable risk tools. For scoring details, interobserver variability, and integration with prediction models, see Section 5.2 Risk Stratification Tools.

### 4.4. Histologic Mimics and Differential Diagnosis

Mesangial IgA-dominant staining supports IgAN but is not pathognomonic; clinicopathologic correlation and exclusion of secondary causes and mimics are essential [60,65]. Machine learning models are emerging as adjuncts to help differentiate primary IgAN from secondary IgA-dominant forms using clinical features [75,76].

Table 1 summarizes the major histologic mimics, key clinical–pathologic differentiators, and management pitfalls.

Initial evaluation should be guided by clinical context and may include liver function tests (LFTs); hepatitis B virus (HBV)/hepatitis C virus (HCV)/human immunodeficiency virus (HIV) serologies; complement (C3/C4); antinuclear antibody (ANA)/antineutrophil cytoplasmic antibody (ANCA); and inflammatory markers, including C-reactive protein (CRP), with cultures and imaging when infection is suspected [77]. Suspected monoclonal disease warrants serum and urine immunofixation and serum free light chains. Management should target the systemic driver first, reserving immunosuppression for confirmed primary IgAN [77,78].

**Table 1:** Primary IgAN vs. common mimics: clinical and pathologic differentiators.

Entity	Typical Context	Immunofluorescence (IF)	Electron Microscopy (EM)	Key Labs/Serology	Management/Pitfalls
Primary IgAN	Microscopic hematuria ± sub-nephrotic proteinuria; synpharyngitic; no systemic features.	Mesangial IgA dominant/co-dominant; C3 common; C1q minimal/absent.	Mesangial ± paramesangial electron-dense deposits (EDD).	Exclude secondary causes; ANA/ANCA typically negative.	Apply MEST-C score; supportive care ± immunosuppression
IgA Vasculitis Nephritis (IgAV)	Palpable purpura, arthralgia, abdominal pain; often pediatric.	IgA-dominant; Crescents and Endocapillary hypercellularity more frequent than IgAN [79].	Mesangial ± subendothelial EDD; crescents common.	Skin biopsy (dermal IgA); systemic features guide diagnosis.	Treat as vasculitis; immunosuppression per systemic/renal activity.
IgA-Dominant Infection-Related GN (IgA-IRGN)	Older/diabetic/immunocompromised; staphylococcal/streptococcal infection (occult or recent).	C3-dominant or strong C3 with IgA; capillary wall deposits [80].	Subepithelial “humps” ± subendothelial EDD [81].	Low serum C3, normal C4; ↑ CRP; culture/imaging for infection.	Eradicate infection first; avoid empiric immunosuppression—misdiagnosis worsens outcomes.
PGNMID-IgA/MGRS	Possible plasma cell/lymphoid clone; nephritic–nephrotic; MPGN-like pattern.	Monotypic IgA (κ or λ light chain restriction) [77].	Granular mesangial + capillary wall EDD.	Serum/urine immunofixation; free light chains; hematology consult.	Clone-directed therapy (chemotherapy); not standard IgAN therapy.
Liver Disease–Related IgA	Cirrhosis/portal hypertension; edema/ascites; alcohol use.	Diffuse mesangial polyclonal IgA with C3.	Mesangial EDD.	Elevated LFTs; polyclonal ↑ serum IgA.	Treat liver disease; immunosuppression generally not indicated.
Celiac/IBD-Associated IgA	GI symptoms; malabsorption; known IBD history.	Mesangial IgA; Kidney IgA-TG2 co-deposits are specific for Celiac [82].	Mesangial EDD.	tTG-IgA serology; duodenal biopsy; IBD evaluation.	Treat mucosal driver (gluten-free diet/IBD therapy); causal link established [83].

Abbreviations: tTG (tissue transglutaminase), MPGN (membranoproliferative glomerulonephritis), MGRS (monoclonal gammopathy of renal significance), PGNMID (proliferative glomerulonephritis with monoclonal immunoglobulin deposits), IBD (inflammatory bowel disease).

### 4.5. Pediatric vs. Adult Presentation

IgAN occurs across all ages, but pediatric- and adult-onset disease differ in presentation, biopsy triggers, histopathology, and clinical trajectory, informing timely diagnosis, risk stratification, and individualized management [60,84]. In terms of presentation and diagnostic pathways, children more often present with episodic gross hematuria around upper respiratory infections and are detected earlier through symptoms or East Asian school screening, whereas adults more commonly present with persistent urinary abnormalities. These differences contribute to variation in stage at diagnosis, and adolescents and young adults remain particularly vulnerable to transition-of-care gaps [60,85,86,87]. Recent IPNA guidance further clarifies that kidney biopsy is recommended in children with persistent or recurrent hematuria accompanied by repeated UPCR > 0.5 mg/mg, and should also be considered promptly when hematuria is associated with nephrotic-range proteinuria and/or reduced eGFR, with weaker support even at lower but persistent proteinuria levels (UPCR 0.2–0.5 mg/mg) [86].

Histopathologically, findings are typically more active in children (M1/E1) and more chronic in adults (S1, T1/T2), broadly consistent with VALIGA; however, pediatric prognostic performance of MEST-C remains less certain and is further limited by interobserver variability [63,65,85].

Therapeutically and longitudinally, children more often receive earlier corticosteroids or other immunosuppression, whereas adults emphasize optimized supportive care with immunosuppression reserved for high-risk disease. Recent pediatric guidance also supports a biopsy-informed, risk-adapted approach in which optimized supportive care remains foundational, while immunosuppression is individualized because high-quality pediatric trial data remain limited [86]; although proteinuria may remit faster in pediatric cohorts, relapses and cumulative toxicity shape long-term outcomes, supporting lifelong surveillance and structured transition to adult care alongside pediatric-focused validation of prognostic models and biomarker integration [61,84,85,86,87,88,89].

## 5. Risk Stratification and Biomarkers

### 5.1. Clinical Risk Predictors

Accurate risk stratification in IgAN is anchored in readily available clinical variables—particularly proteinuria, eGFR, and blood pressure—that independently predict long-term outcomes. These core predictors underpin validated risk tools such as the IIgAN-PT, which integrates clinical and histologic data to estimate the risk of kidney function decline and has been used to enrich clinical trial cohorts for higher-risk patients [90,91,92].

Persistent microscopic hematuria may also provide adjunct information on disease activity and prognosis when interpreted alongside proteinuria and eGFR. Recent data suggest that baseline microhematuria correlates with active histologic lesions, particularly M1, E1, and C lesions, and that greater microhematuria during follow-up is independently associated with faster eGFR decline even after adjustment for proteinuria and T score, supporting its role as a complementary longitudinal marker rather than a standardized standalone risk tool, although measurement variability and non-glomerular sources still limit routine implementation [93,94]. Proteinuria is the strongest clinical predictor of progression; baseline and time-averaged levels consistently associate with adverse kidney outcomes. Reflecting a major shift in management, the KDIGO 2025 guidelines have lowered the treatment target from <1 g/day to <0.5 g/day (ideally <0.3 g/day) [20,62]. Sustained elevations increase the likelihood of kidney failure requiring kidney replacement therapy and all-cause mortality [20,95]. In a nationwide Japanese cohort, early dipstick proteinuria improvement at about 9 months predicted slower eGFR decline and fewer major kidney events [96].

eGFR adds complementary prognostic information; in particular, lower baseline eGFR predicts higher long-term risk across cohorts. eGFR slope is also widely used as a surrogate endpoint in clinical trials and is associated with long-term kidney outcomes [62,95,97]. In a CT- and biopsy-based cohort, higher single-nephron eGFR was associated with faster kidney function decline, suggesting nephron-level hyperfiltration may refine risk assessment beyond eGFR cutoffs alone [98].

Blood pressure remains an independent modifiable target; elevated blood pressure at diagnosis associates with faster functional decline. Current guidelines advocate for a stricter systolic target of <120 mm Hg to maximize renal and cardiovascular protection [62,65].

Sociodemographic and health-system factors also modify risk. In the large US CURE-CKD registry, lack of commercial insurance and medication non-adherence were independently linked to Major Adverse Kidney Events (MAKE) [97]. Older age at diagnosis is also associated with higher long-term risk [95].

### 5.2. Histopathologic Risk Stratification: Oxford Classification and MEST-C Scoring

In contemporary multiethnic validation cohorts, among MEST-C components, chronicity markers (S and, particularly, T) show the strongest and most reproducible associations with kidney failure and retain independent prognostic value in multivariable models. Machine learning analyses further highlight T as a dominant determinant of long-term kidney survival, emphasizing the weight of tubulointerstitial injury over inflammatory activity for hard endpoints [73,99]. Complementing these chronicity signals, a nationwide multicenter registry analysis suggested that the ratio of globally sclerotic glomeruli on diagnostic biopsy may offer additional quantitative stratification for 5-year adverse kidney outcomes, although external validation is required before routine clinical adoption [100]. Consistent with this emphasis on chronic injury, pragmatic prognostic nomograms retain Oxford T as a core predictor alongside routine clinical variables [101].

Active inflammatory lesions (M, E, C) display more variable predictive value, in part because prognostic impact is modified by immunosuppressive therapy. The prognostic significance of crescents is nuanced: C1 lesions (<25% crescents) may be more responsive to immunosuppression, whereas extensive crescents (C2) are consistently associated with poorer prognosis and often co-occur with necrosis, supporting consideration of more intensive therapy [102,103].

Integrating MEST-C with clinical variables markedly enhances predictive power. The IIgAN-PT, which combines histology with eGFR, proteinuria, and blood pressure, has historically achieved superior accuracy compared to clinical variables alone [90]. However, its application is evolving: recent validation suggests potential risk overestimation in contemporary cohorts treated with newer non-immunosuppressive therapies (e.g., sodium-glucose co-transporter 2 (SGLT2) inhibitors), deep learning approaches may reduce inter-observer variability affecting E and C scoring, and non-invasive adjuncts (clinical-only models and urinary fibrosis biomarkers, such as epidermal growth factor and MCP-1) may support stratification when biopsy is not feasible [104,105,106,107].

The KDIGO 2025 guidelines emphasize that while MEST-C is essential for staging, it should not dictate treatment in isolation and is best interpreted alongside clinical variables and integrated prediction tools, although risk stratification frameworks are not fully uniform across published guidance [62].

### 5.3. Individualized Risk Prediction Models and Tools

The IIgAN-PT is a widely used, internationally validated prognostic tool for IgAN risk stratification. It integrates eGFR, proteinuria, mean arterial pressure (MAP), and Oxford MEST scores to estimate the risk of a ≥50% eGFR decline or kidney failure over a defined time horizon, although it does not incorporate crescents or hematuria, with extensive international validation [90]. Longitudinal validation studies support sustained discrimination, and recent analyses suggest that removing race has minimal impact on model performance in some cohorts, supporting race-neutral application where appropriate [91]. Beyond this static baseline framework, emerging approaches incorporate longitudinal clinical data to generate time-updated risk trajectories, whereas machine-learning methods have been explored to reduce inter-observer variability in histology-derived features or support risk estimation when biopsy data are unavailable, but these approaches remain investigational and require prospective validation before routine clinical use [99,108,109,110]. However, the main practical limitation remains calibration drift in contemporary cohorts receiving newer therapies, suggesting that population-specific recalibration—including in pediatric cohorts—may be needed before broad implementation [90,91,99,101,104,108,110,111].

### 5.4. Emerging Biomarkers of Disease Activity and Progression

Candidate biomarkers are being investigated to complement clinical variables and histology, with the goal of improving earlier risk detection and dynamic monitoring in IgAN [61,112]. Table 2 summarizes the clinically relevant predictors and biomarkers highlighted in this section. However, current guideline-supported decision-making still relies primarily on established clinical measures—especially proteinuria—while these biomarkers remain investigational and have not yet been prospectively validated for routine clinical use.

Among urinary biomarkers, urinary IL-6 and the EGF/MCP-1 ratio have been associated with disease activity and independently predict progression, with the EGF/MCP-1 ratio also correlating with tubulointerstitial fibrosis as a non-invasive estimate of the T component [107,113,114]. Urinary CA1 tracks microscopic hematuria and is measurable in stored samples, while urinary leukemia inhibitory factor normalized to creatinine (uLIF/Cr) has been associated with prognosis and glucocorticoid response, supporting potential roles in standardized monitoring rather than established clinical decision-making [115,116].

Serologic and tissue biomarkers may provide complementary pathobiologic information. Elevated Gd-IgA1 and CICs correlate with mesangial deposition and disease activity, and early reductions in Gd-IgA1 and poly-IgA during Nefecon therapy have been linked to subsequent proteinuria improvement, suggesting potential value as dynamic monitoring markers rather than routine tools [26,117]. By contrast, the serum IgA/C3 ratio has shown inconsistent performance across cohorts [118,119]. At the tissue level, glomerular C4d deposition is prognostically adverse but not guideline-endorsed for therapeutic decision-making, whereas in situ assessment of glomerular C3/C5 convertases, KM55 immunohistochemistry, and tubulointerstitial CD68^+^ macrophage burden remain promising but incompletely validated adjuncts [62,67,120,121].

Transcriptomic and multi-omic profiling may further refine mechanistic stratification. Multi-omic network biomarkers (KMN) have shown prognostic discrimination exceeding that of the IIgAN-PT, and integrative analyses have identified ACOX2 as a candidate protective marker, but these approaches remain limited by cost, complexity, and the need for broader standardization and external validation before clinical adoption [112,122,123,124,125].

**Table 2:** Clinically relevant predictors and biomarkers in IgAN.

Biomarker/Class	Sample Type	Prognostic/Diagnostic Role	Key References
Proteinuria (time-averaged)	Urine	Strongest predictor of progression; remission associates with favorable outcomes	[61,63,65]
Persistent microscopic hematuria	Urine	Adjunct marker of disease activity and prognosis; linked to active lesions and faster eGFR decline, but not standardized as a standalone risk tool	[94]
eGFR (baseline/slope)	Blood (serum creatinine-based)	Predicts risk of kidney failure; slope is a regulatory-accepted surrogate in trials	[90,114]
Oxford MEST-C	Kidney biopsy	Histologic scores predict outcomes	[65,90]
Gd-IgA1 and CICs	Serum/urine	Dynamic markers; early reduction predicts therapeutic response to Nefecon	[26,117]
Urinary IL-6	Urine	Independent predictor of progression in cohort studies	[113,114]
EGF/MCP-1 ratio	Urine	Associated with tubular damage; non-invasive correlate of fibrosis/T component	[107,114]
Urinary carbonic anhydrase 1 (CA1)	Urine	Quantitative marker of microscopic hematuria; measurable in stored samples	[116]
Novel tissue markers	Kidney biopsy	C3/C5 convertases indicate ongoing complement activation	[121]
Novel markers (very late antigen-4 [VLA-4], vascular endothelial growth factor-D [VEGF-D])	Serum	Reported higher diagnostic accuracy than Gd-IgA1; emerging prognostic role	[126]
KM55 (mesangial Gd-IgA1 immunohistochemistry)	Kidney biopsy	Biopsy-based diagnostic adjunct (suggested cutoff ≥2+); imperfect specificity requires clinicopathologic correlation; prognostic association reported but not independent after Oxford adjustment.	[66,67,68]

### 5.5. Genetic and Multi-Omics Risk Stratification

Risk prediction in IgAN is increasingly incorporating genetic and multi-omics frameworks to capture disease heterogeneity and complement proteinuria- and eGFR-based tools. Polygenic risk scores (PRS) may extend stratification beyond routine clinical variables; a trans-ethnic PRS based on 30 independent risk loci stratified lifetime disease risk across European and East Asian populations, and higher genetic risk scores were associated with earlier onset, gross hematuria, and active (M/E) lesions, supporting enrichment for an immunologically “active” phenotype [57,58]. Multi-omic and AI-driven models may further refine molecular risk groups, with network biomarker, transcriptomic, and integrated clinical–histologic models showing encouraging prognostic performance in research cohorts [124,125,127]. However, these approaches remain investigational and will require prospective external validation, feasibility assessment, and context-specific calibration before broad clinical implementation [109,112].

### 5.6. Risk Stratification in Pediatric and Transplant IgAN

Risk stratification in pediatric and transplant IgAN requires context-specific interpretation, as adult-derived tools may not fully generalize across age, event rates, and treatment effects; in transplant recipients, recurrent IgAN in the allograft represents an important disease-specific risk domain; MEST-C can remain informative but should be applied after clinicopathologic confirmation and interpreted within these constraints [65,84,128,129]. Tool-level inputs and performance summaries are provided in Table 3.

In children, adverse outcomes are associated with proteinuria burden and eGFR trajectory, alongside histologic features including M and T (and, in some cohorts, S and extensive crescents [C2]); a subset may show rapid early decline, supporting closer longitudinal monitoring, while omics panels remain investigational [65,112,128,129,130,131].

In transplantation, recurrent IgAN is a major contributor to graft dysfunction and loss; reported risk factors include younger recipient age, faster pre-transplant progression to end-stage kidney disease (ESKD), active native kidney lesions (e.g., crescents), and serologic signals (elevated Gd-IgA1 and anti-Gd-IgA1), with donor and HLA-related features variably implicated across cohorts. Distinguishing recurrent from de novo IgAN remains essential because management and prognostic implications differ [61,132,133,134,135,136].

In practice, risk assessment prioritizes longitudinal clinical course and histology (when available), with biomarkers/omics used as adjuncts as validation matures. Table 3 summarizes available tools and their performance [109,112,124,131,137].

## 6. Current Management (Conventional Therapy)

### 6.1. Supportive Care and Risk Factor Control

Reducing proteinuria remains the primary modifiable target in IgAN. KDIGO 2025 emphasizes optimized supportive care—RAS blockade, SGLT2 inhibitors, and lifestyle modification—as foundational therapy and, in high-risk patients, recommends initiating these measures alongside disease-modifying treatments rather than as a sequential “pre-immunosuppression” phase [62,138]. A systematic review of 76 randomized controlled trials reported near-universal inclusion of proteinuria outcomes and frequent assessment of eGFR slope, reinforcing these endpoints as central to supportive therapy evaluation [139]. Although proteinuria remains the principal treatment target, persistent microscopic hematuria may also reflect ongoing disease activity, and remission of hematuria has been associated with improved renal outcomes; however, it is not yet used as a standardized standalone treatment target [94]. Accordingly, maximizing a nephroprotective “bundle” can meaningfully reduce proteinuria and attenuate eGFR decline, establishing supportive care as the platform on which subsequent therapies build [139,140]. In practice, treatment sequencing is anchored in optimized supportive care for all patients, with subsequent escalation guided by persistent proteinuria, overall risk, drug availability, and toxicity profile rather than by a fixed universal algorithm.

#### 6.1.1. Blood Pressure Control and RAS Inhibition

Current guidelines recommend a strict systolic blood pressure target of <120 mm Hg (where tolerated) and the maximal tolerated dose of an ACEi or ARB for patients with proteinuria ≥ 0.5 g/day [62]. This intensive target is supported by cohort data showing improved kidney survival and fewer adverse outcomes with systolic BP < 120 mm Hg, when compared with standard targets [141].

While dual RAS blockade (ACEi + ARB) is discouraged because of hyperkalemia and acute kidney injury risk, the therapeutic landscape has expanded to include dual endothelin and angiotensin receptor antagonists (DEARA). Sparsentan, a first-in-class DEARA, combines hemodynamic RAS blockade with endothelin inhibition to enhance antiproteinuric efficacy (efficacy and safety are reviewed in Section 7.3) [62,138]. Accordingly, risk factor control combines intensive hemodynamic management with agents that stabilize kidney function [141,142].

#### 6.1.2. Dietary Sodium Restriction and Non-Pharmacologic Measures

Dietary sodium restriction is a potent, independent tool for risk reduction in IgAN. KDIGO 2025 recommends limiting sodium intake to <2 g/day (<5 g salt/day) for all patients [62]. In the LowSALT CKD trial, sodium restriction lowered blood pressure and proteinuria and reduced extracellular fluid volume, enhancing the antiproteinuric effect of concomitant RAS blockade [62,143].

Because adherence to low-sodium diets varies, structured counseling is essential. Beyond sodium restriction, weight optimization (BMI < 25 kg/m^2^) and smoking cessation are core elements of comprehensive supportive care, as applied in STOP-IgAN; observational data link obesity and smoking to faster progression, reinforcing lifestyle modification as integral to management [140,142].

#### 6.1.3. Sodium–Glucose Co-Transporter 2 (SGLT2) Inhibitors

SGLT2 inhibitors are now a core component of optimized supportive care for IgAN and are recommended for eligible patients in addition to maximal RAS blockade and lifestyle measures. In the prespecified IgAN subgroup of DAPA-CKD (n = 270; 94% biopsy-proven), dapagliflozin significantly reduced the risk of the primary composite outcome (sustained ≥50% eGFR decline, end-stage kidney disease, or renal/cardiovascular death) versus placebo (hazard ratio 0.29, 95% CI 0.12–0.73) [144].

These findings are supported by broader CKD evidence, including EMPA-KIDNEY and the SMART-C meta-analysis, which show consistent reductions in kidney disease progression with SGLT2 inhibition across etiologies and independent of diabetes status [145,146]. However, emerging observational data suggest that the antiproteinuric response to SGLT2 inhibitors may vary by body habitus, with a weaker short-term response reported in patients with BMI < 25 kg/m^2^; however, given the mixed cohort and limited follow-up, this signal remains hypothesis-generating rather than practice-changing [147]. Although IgAN-focused randomized trials remain ongoing to refine long-term safety in younger populations, current guidance supports SGLT2 inhibitors as standard nephroprotection for adults with IgAN at risk of progression [148,149]. In real-world practice, adjunct finerenone has been associated with higher proteinuria complete response (<0.5 g/day) and fewer ≥30% eGFR-decline events, with a stronger remission signal when combined with SGLT2 inhibitors and no apparent increase in serum potassium [150].

### 6.2. Corticosteroid Therapies in IgAN

#### 6.2.1. Systemic Corticosteroids

Systemic corticosteroids remain a key option in selected high-risk IgAN patients with persistent proteinuria (>0.75–1 g/day) despite optimized supportive care. However, contemporary guidance has shifted toward cautious, risk-stratified use. KDIGO 2025 recommends systemic steroids primarily when TRB is unavailable or contraindicated, given the systemic toxicity associated with prolonged glucocorticoid exposure [62,151,152].

The evidence base highlights a trade-off between renal protection and toxicity. A recent meta-analysis of placebo-controlled phase 2b/3 IgAN trials reinforced this balance, showing that corticosteroids are among the most effective classes for reducing proteinuria and improving eGFR slope, but also carry the clearest serious adverse event burden, particularly at higher doses [153]. Against this broader context, the earlier STOP-IgAN trial showed that a 6-month steroid course reduced proteinuria transiently but failed to improve long-term kidney survival while increasing adverse events [140]. Conversely, the definitive TESTING trial demonstrated that oral methylprednisolone significantly reduced major kidney outcomes by approximately 47% (HR 0.53). Crucially, the trial validated a reduced-dose regimen (0.4 mg/kg/day, tapered over 6–9 months) combined with antimicrobial prophylaxis (Pneumocystis prevention), which retained renal benefits while reducing serious adverse events by nearly half [154,155]. A more critical comparison of the TESTING regimens suggests that full-dose methylprednisolone carried the highest toxicity burden, whereas the reduced-dose regimen improved the benefit–risk balance; however, because these regimens were not compared in a head-to-head randomized fashion, the available data support dose minimization as a safety strategy rather than definitive efficacy equivalence between low- and high-dose approaches [153,154,155].

KDIGO 2025 suggests starting methylprednisolone at 0.4 mg/kg/day (maximum 32 mg/day) for 2 months, followed by a slow taper (4 mg/month reduction) [62]. Patient selection is critical; a TESTING secondary analysis modeling individualized treatment benefit demonstrated wide heterogeneity in predicted absolute risk reduction, supporting steroid targeting to those most likely to benefit, alongside the observed gradient of greater benefit at higher baseline proteinuria and lower eGFR, provided infection risk is managed [155,156,157].

Regional approaches differ significantly. While Western guidance favors the oral tapering regimen described above, Japanese guidelines and recent prospective data support intravenous steroid pulse therapy, often combined with tonsillectomy. In a large nationwide cohort (n = 941), this combination was associated with a lower risk of kidney events compared with non-steroid therapy, suggesting potentially distinct therapeutic patterns in Asian populations [158].

To mitigate toxicity further, steroid-sparing combinations are under investigation. In a propensity-score-matched cohort, adjunct hydroxychloroquine with systemic glucocorticoids was associated with a higher likelihood of successful steroid tapering and greater short-term proteinuria reduction compared with glucocorticoids alone [159]. A 2024 meta-analysis found that low-dose corticosteroids combined with leflunomide reduced proteinuria and stabilized serum creatinine compared with full-dose steroids alone, offering a potential alternative for patients intolerant to high-dose glucocorticoids [160].

#### 6.2.2. Targeted-Release Budesonide (Nefecon)

TRB is formulated to deliver budesonide to the distal ileum, maximizing exposure to Peyer’s patches and gut-associated lymphoid tissue (GALT). This formulation was designed to modulate mucosal immune activity relevant to IgAN pathogenesis; however, a specifically localized mechanism remains unproven, and some systemic absorption and systemic steroid effects still occur [8,161].

In the Phase 2b NEFIGAN trial [161] and the Phase 3 NefIgArd program [8], TRB reduced proteinuria and preserved eGFR versus placebo, with sustained benefit on longer-term follow-up (Table 4). TRB is now approved by the U.S. Food and Drug Administration to reduce the loss of kidney function in adults with primary IgAN at risk of disease progression [162]. Real-world reports, including in patients with severe renal impairment (eGFR < 30 mL/min/1.73 m^2^), suggest effectiveness and tolerability outside clinical trial settings.

Indirect comparisons suggest that TRB may have a more favorable tolerability profile than systemic corticosteroids, with fewer severe infections and metabolic adverse events, although mild steroid-related effects (e.g., acne, edema) still occur. However, no head-to-head trials have compared TRB with reduced-dose systemic corticosteroids, and available comparisons remain cross-trial; therefore, superior efficacy or safety versus low-dose systemic steroids cannot be concluded [153,161,163]. Accordingly, KDIGO 2025 and other expert algorithms generally position Nefecon as an option to consider before systemic corticosteroids in selected high-risk patients, when available, rather than as a proven superior alternative.

**Table 4:** Corticosteroid trials in IgAN (systemic and gut-targeted).

Trial (Year)	Population and Intervention	Main Findings	Safety
STOP-IgAN (2015) [140]	Adults; 6-month systemic corticosteroids after optimized supportive care run-in	No sustained benefit on eGFR outcomes vs. supportive care alone	Significant increase in infections, weight gain, and glucose intolerance
TESTING (2022) (initial full-dose arm; protocol stopped early) [154]	High-risk IgAN; methylprednisolone (full-dose arm)	Significantly reduced risk of composite kidney failure event (HR 0.53)	High rate of serious adverse events (SAEs) in full-dose arm led to protocol change
TESTING (2022/24) (revised reduced-dose + *Pneumocystis prophylaxis*) [154,164]	High-risk IgAN; methylprednisolone 0.4 mg/kg/day + antimicrobial prophylaxis	Consistent benefit on kidney failure (HR ~0.51) and proteinuria reduction	SAEs were reduced versus the earlier full-dose TESTING experience, with dose reduction and antimicrobial prophylaxis improving the safety profile
NEFIGAN (2017) [161]	Phase 2b; TRB 16 mg/day vs. placebo (9 months)	Significant reduction in proteinuria; stabilization of eGFR	SAEs similar to placebo; mild steroid-related AEs (acne, edema)
NefIgArd (2023) [163]	Phase 3; TRB 16 mg/day (9 months) with 2-year follow-up	Slower eGFR decline (−2.47 vs. −7.52 mL/min/1.73 m^2^ over 2 years); 27% proteinuria reduction	Generally well tolerated; SAE rate was comparable to placebo in the trial, with no severe infections reported

#### 6.2.3. Alternative Oral Budesonide Formulations When Nefecon Is Unavailable

TRB (Nefecon/Tarpeyo) is the only oral budesonide formulation currently approved by the U.S. Food and Drug Administration for primary IgAN at risk of disease progression, and should be preferred when available [165]. When TRB is not accessible, other enteric/modified-release budesonide products have been used off-label; however, formulations differ materially in enteric polymers, pH thresholds, and primary release sites and therefore should not be assumed to be interchangeable in IgAN [166]. Key formulation-driven differences relevant to off-label selection are summarized in Table 5.

Evidence for non-TRB formulations is limited and heterogeneous, consisting largely of observational cohorts reporting proteinuria reductions with generally stable kidney function [167,168,169]. A controlled trial of a non-TRB controlled-release/enteric-coated budesonide capsule also reported additional proteinuria reduction versus standard care with preserved eGFR during follow-up [170]. Given formulation-specific delivery and a smaller evidence base than the TRB trial program, use of non-TRB budesonide should be framed explicitly as off-label, with careful monitoring for glucocorticoid effects and drug–drug interactions [166,171].

**Table 5:** Formulation-driven differences among oral budesonide products relevant to off-label use in IgAN.

Product	Platform	Enteric Trigger (Reported)	Primary Release Region (Reported)	IgAN Evidence in This Manuscript Set	Exposure/Safety Note (Documented)
Budenofalk^®^	Enteric-coated beads/capsules	~6.4	Ileum and ascending colon	Yes—cohort/observational	Systemic effects possible; cohorts report generally mild events
Entocort^®^ EC	Enteric-coated beads	>5.5	Ileum and ascending colon (more proximal start)	No (IgAN-specific outcomes not included in this manuscript set)	Label/PK highlights adrenal suppression and CYP3A4 interaction risk
Cortiment^®^ (MMX)	MMX tablet	~7.0	Colon	No	Primarily colonic delivery; IgAN data absent
Nefecon (reference)	Targeted-release beads/capsule	Proprietary	Distal ileum (GALT-focused targeting)	Yes—TRB trial program (covered in Section 6.2.2); FDA approved	Lower dose-adjusted cortisol suppression vs. Entocort in comparative PK

**Footnote (Table 5):** *Sources for formulation/release-site and PK/safety notes:* [162,166,171,172]. *Sources for IgAN outcome evidence referenced in the table:* [168,169,170].

### 6.3. Immunosuppressive Agents

Non-biologic immunosuppression is generally reserved for selected patients after optimized supportive care and after considering systemic corticosteroids or TRB; efficacy positioning is summarized here, with safety in Section 6.4.

#### 6.3.1. Mycophenolate Mofetil (MMF)

The KDIGO 2025 guidelines discourage the routine use of MMF in IgAN but allow its consideration as a glucocorticoid-sparing option specifically in Chinese patients, noting that evidence for efficacy in non-Chinese populations remains insufficient [62].

This recommendation reflects conflicting trial data. In Chinese cohorts, several randomized studies have suggested benefit, although results have not been uniformly positive across all settings. In Hong Kong, MMF improved persistent proteinuria in selected patients despite optimized renin–angiotensin system blockade, and longer-term follow-up suggested slower kidney function decline and better renal survival despite attenuation of the early antiproteinuric effect over time [173]. In active proliferative IgAN, MMF combined with low-dose prednisone achieved similar remission efficacy to full-dose prednisone with fewer steroid-related adverse events, supporting a steroid-sparing role in selected patients [174]. More recently, the MAIN trial showed that MMF added to optimized supportive care reduced CKD progression and composite kidney outcomes in progressive IgAN [175]. In contrast, earlier Western trials failed to demonstrate renal benefit compared to placebo or supportive care [176,177]. Recent retrospective analyses from Western centers, such as the Mayo Clinic, continue to explore MMF’s role as a steroid-sparing adjunct, though results have not yet provided sufficient evidence to overturn current guideline restrictions [178].

Interest in optimizing this pathway persists. Ongoing investigations, such as the EMSAR-IgAN trial, are evaluating enteric-coated mycophenolate sodium (EC-MPS) in combination with hydroxychloroquine, reflecting a shift toward selective, multi-target immunomodulation to minimize toxicity [179]. In clinical practice, MMF remains a second-line consideration, primarily for patients who require immunosuppression but have contraindications to high-dose corticosteroids, while its role outside Chinese populations remains uncertain [62,180].

#### 6.3.2. Cyclophosphamide

Cyclophosphamide (CYC) is no longer recommended for routine use in IgAN, but remains a rescue therapy for select high-risk presentations. KDIGO 2025 restricts CYC—typically in combination with systemic corticosteroids—to patients with RPGN (defined as a ≥50% decline in eGFR over ≤3 months) or pulmonary–renal syndrome; in these fulminant cases, the guidelines advise following cyclophosphamide-based induction protocols used for ANCA-associated vasculitis [62].

This targeted approach reflects a dichotomy in the evidence base. Early studies in aggressive, crescentic, or rapidly deteriorating disease suggested that CYC-based regimens could stabilize renal function and induce remission [181,182,183], and a recent retrospective analysis similarly reported improved kidney survival with CYC plus steroids versus supportive care in progressive disease [184]. In contrast, in broader populations with moderate risk or slower progression, CYC did not demonstrate sustained renal benefit and was associated with substantially increased toxicity, including infection risk [140]. Consequently, its role is now confined to the most aggressive phenotypes, where potential organ salvage outweighs cytotoxicity risk [62].

#### 6.3.3. Calcineurin Inhibitors (CNIs)

Tacrolimus and cyclosporine can rapidly lower proteinuria but are not disease-modifying; relapse after withdrawal is common, and nephrotoxicity limits duration. Randomized and cohort studies show short-term antiproteinuric effects without sustained eGFR benefit [185,186,187]. Consequently, KDIGO 2025 does not recommend CNIs in adults given the lack of proven long-term efficacy, although they may remain a second-line consideration in children or in specific regional practices [62].

#### 6.3.4. Other Non-Biologic, Non-CNI Immunosuppressive Agents

Leflunomide has emerged as a potential steroid-sparing agent, primarily within East Asian practice. A 2024 systematic review and multiple randomized trials indicate that leflunomide combined with low-dose corticosteroids reduces proteinuria and stabilizes kidney function comparably to full-dose systemic corticosteroids, but with a significantly lower incidence of serious adverse events [160,188,189]. However, validation in non-Asian populations remains limited.

By contrast, older antimetabolites such as azathioprine and mizoribine are generally not recommended for routine use. Network meta-analyses suggest limited efficacy and inconsistent kidney survival benefits compared with current standard-of-care options, restricting these agents to niche second-line roles in select regional guidance [62,190].

### 6.4. Treatment-Associated Risks

Immunosuppressive therapy in IgAN requires balancing efficacy with toxicity; this subsection summarizes key adverse-effect profiles and practical monitoring considerations.

#### 6.4.1. Corticosteroid-Related Toxicity

Systemic corticosteroids carry a well-documented burden of metabolic and infectious risks. Common metabolic complications include weight gain, dysglycemia, and hypertension. In STOP-IgAN, a 6-month systemic corticosteroid regimen increased infections and glucose intolerance [140]. In TESTING, the original higher-dose regimen was stopped early due to excess fatal infections; the revised reduced-dose regimen with Pneumocystis prophylaxis improved safety, supporting dose minimization and antimicrobial prophylaxis as key mitigation strategies [62,154].

#### 6.4.2. Safety of Targeted-Release Budesonide (Nefecon)

Designed to minimize systemic exposure via high first-pass metabolism, TRB exhibits a more favorable safety profile than systemic corticosteroids. While not entirely free of systemic effects—mild to moderate acne, peripheral edema, and hypertension are frequently reported—serious adverse events in Phase 3 trials were comparable to placebo (including no increase in serious infections) [8,62]. This supports its preferential use in patients at higher risk of systemic glucocorticoid toxicity.

#### 6.4.3. B-Cell Immunomodulation (Telitacicept)

In a real-world cohort, telitacicept was not associated with serious adverse events, with low reported infection rates [191].

#### 6.4.4. Non-Steroidal Immunosuppressants

MMF can be considered a second-line, glucocorticoid-sparing option in selected settings, but requires routine monitoring for gastrointestinal intolerance, cytopenias, and infection risk [62,180]. Ongoing EC-MPS-based strategies are being evaluated to minimize toxicity while maintaining efficacy [179].

Regarding CNIs, nephrotoxicity and frequent proteinuria relapse after discontinuation limit the duration and complicate their long-term use [185,186,187].

#### 6.4.5. Risk Mitigation Strategies

Effective risk management involves selecting the lowest effective dose and implementing antimicrobial prophylaxis when using systemic corticosteroids, alongside structured surveillance for metabolic complications (glucose, blood pressure, and weight) and infection risk [62,154].

### 6.5. Tonsillectomy and Other Local Therapies

KDIGO 2025 acknowledges the widespread use of tonsillectomy in Japan but does not recommend routine adoption in other regions, citing a lack of randomized evidence in non-Asian populations [62]. Accordingly, tonsillectomy is not included in the IIgAN-PT [62,90].

#### 6.5.1. Mechanistic Rationale and Japanese Evidence

The procedure targets the palatine tonsils as a source of aberrant mucosal immune responses and pathogenic Gd-IgA1 production [180]. In Japanese cohorts, tonsillectomy combined with steroid pulse therapy (TSP) has consistently demonstrated higher clinical remission rates and better kidney survival compared to tonsillectomy alone or systemic steroid therapy [192,193,194]. A 2024 retrospective analysis further supported superior renal protection with TSP versus tonsillectomy monotherapy, suggesting synergy between mucosal antigen elimination and systemic immune suppression [195].

#### 6.5.2. Patient Selection and Future Outlook

Recent data have refined patient selection. In a nationwide cohort analysis, the renal benefit of TSP was most pronounced in patients with moderate to severe proteinuria (≥1.0 g/day) and active histological lesions, whereas benefit in mild disease was less clear [196]. In clinical practice, tonsillectomy—typically as TSP—remains a standard-of-care option in Japan and may be considered in other experienced centers for carefully selected high-risk patients; however, broader global adoption awaits multinational randomized trials. As mechanism-based systemic therapies targeting the gut–kidney axis (e.g., Nefecon, APRIL inhibitors) become widely available, reliance on surgical interventions may diminish [180,190].

## 7. Emerging Therapies and Clinical Trials

Building on the mechanistic framework in Section 3, this section reviews emerging therapies aligned to pathogenic targets and summarizes the key clinical trial signals across programs.

### 7.1. B-Cell and APRIL/BAFF Pathway Inhibitors

BAFF/APRIL pathway inhibition aims to reduce pathogenic Gd-IgA1 production by limiting B-cell and plasma cell survival.

#### 7.1.1. Dual Inhibition (BAFF/APRIL)

Atacicept: In the Phase 3 ORIGIN 3 trial, atacicept, a fusion protein inhibiting both BAFF and APRIL, met its primary endpoint by reducing proteinuria versus placebo at week 36, while also showing stabilization of eGFR and substantial reductions in Gd-IgA1 and hematuria [197].

Telitacicept: This fusion protein demonstrated efficacy in a Phase 2 trial (n = 44), resulting in a 49% reduction in proteinuria and eGFR preservation over 24 weeks [198]. Real-world cohorts have also reported substantial short-term proteinuria reduction, with supportive improvements in hematuria and generally favorable short-term safety, although interpretation remains limited by non-randomized design and short follow-up [191,199].

#### 7.1.2. Selective APRIL Inhibition

Sibeprenlimab, a humanized monoclonal antibody specifically neutralizing APRIL, offers a more targeted approach. In a Phase 2 trial (n = 155), sibeprenlimab reduced the geometric mean ratio of 24 h proteinuria by 47.2% at 12 months (vs. placebo), with a favorable safety profile [200]. In a prespecified interim analysis of the Phase 3 VISIONARY trial (first 320 randomized), subcutaneous sibeprenlimab achieved a placebo-adjusted 51.2% reduction in 24 h urinary protein-to-creatinine ratio at 9 months with similar serious adverse event rates to placebo, while eGFR-slope outcomes remain pending [201]. Sibeprenlimab has since received U.S. FDA accelerated approval for adults with primary IgAN at risk for disease progression, based on reduction in proteinuria, while confirmatory evidence for long-term clinical benefit remains under evaluation [202].

Zigakibart (BION-1301), another selective APRIL-targeting monoclonal antibody, has also shown promising IgAN-specific activity. In an ongoing phase 1/2 study with 100-week follow-up, zigakibart was well tolerated and was associated with a 60.4% reduction in proteinuria, sustained eGFR stabilization, a notable decrease in hematuria, and rapid durable reductions in IgA, Gd-IgA1, and IgM. Its efficacy and long-term kidney effects are now being evaluated in the ongoing phase 3 BEYOND trial [203]. Selective APRIL blockade may offer a more focused strategy for suppressing pathogenic IgA pathways while preserving broader BAFF-dependent immune function.

#### 7.1.3. Plasma Cell Depletion (Anti-CD38)

Targeting long-lived plasma cells, which may be resistant to conventional B-cell therapies, represents a complementary strategy for refractory disease. Felzartamab, a fully human anti-CD38 monoclonal antibody, was evaluated in the Phase 2a IGNAZ study (n = 54). Treatment with felzartamab for 6 months led to a durable reduction in Gd-IgA1 and stable eGFR, with effects persisting up to 9 months off-therapy. This treatment-free remission potential distinguishes anti-CD38 agents from continuous maintenance therapies, validating CD38 as a high-value therapeutic target [59].

### 7.2. Complement Pathway Inhibitors

Complement inhibitors are being evaluated in IgAN across alternative-, lectin-, and terminal/central-pathway targets to attenuate inflammatory injury [42,204].

#### 7.2.1. Alternative Pathway: Factor B Inhibition

Iptacopan, an oral proximal inhibitor targeting Factor B, showed a 23% reduction in proteinuria in Phase 2 and achieved a 38.3% placebo-adjusted reduction at 9 months in the Phase 3 APPLAUSE-IgAN trial (*p* < 0.0001) [12,205]. Iptacopan has since received U.S. FDA accelerated approval for reduction of proteinuria in adults with primary IgAN at risk of rapid disease progression, while confirmatory evidence for long-term kidney benefit remains under evaluation [206]. The trial is ongoing to determine whether these antiproteinuric effects translate into longer-term eGFR preservation [12].

#### 7.2.2. Lectin Pathway: MASP-2 Inhibition

Narsoplimab (anti-MASP-2) produced early proteinuria reductions, but the Phase 3 ARTEMIS-IgAN study was halted for futility, underscoring that lectin pathway inhibition alone may be insufficient and highlighting the need for biomarker-guided patient selection [138].

#### 7.2.3. Downstream and Central Blockade (C3/C5)

Strategies targeting terminal and central complement components are advancing. Ravulizumab, a long-acting C5 inhibitor, was associated with a 30.1% relative reduction in proteinuria versus placebo in the Phase 2 SANCTUARY trial [207]. Cemdisiran, an RNAi (RNA interference) therapeutic that suppresses hepatic C5 production, achieved a placebo-adjusted geometric mean change in 24 h urinary protein-to-creatinine ratio (UPCR) of −37.4% at week 32 in Phase 2 [42].

Direct IgAN trial evidence for C3 inhibition remains limited; however, data from other complement-mediated glomerulopathies support C3 as a shared convergence node rather than constituting disease-specific proof in IgAN [204]. Consistent with this rationale, in C3 glomerulopathy and primary immune complex membranoproliferative glomerulonephritis, pegcetacoplan reduced proteinuria in the Phase 3 VALIANT trial, providing indirect support for C3 as a therapeutic target across complement-mediated glomerulonephritides [208]. Overall, complement-directed strategies are promising but likely require pathway- and biomarker-informed selection and thoughtful integration with established antiproteinuric therapy and upstream immunomodulation [12,209].

### 7.3. Endothelin Receptor Antagonists and Dual Blockade

ERAs and dual endothelin-A/angiotensin II type 1 (ETA/AT1) blockade provide non-immunosuppressive antiproteinuric therapy on top of optimized RAS inhibition [210,211].

Atrasentan is a highly selective endothelin-A receptor antagonist. In the prespecified interim analysis of the Phase 3 ALIGN study, atrasentan reduced the 24 h urinary protein-to-creatinine ratio by a geometric mean of 38.1% from baseline at week 36, compared with a 3.1% reduction in the placebo group, yielding a significant between-group difference of 36.1 percentage points (95% CI, −44.6 to −26.4; *p* < 0.001) [10]. Based on proteinuria reduction, the U.S. Food and Drug Administration granted accelerated approval of atrasentan (Vanrafia) in 2025 to reduce proteinuria in adults with primary IgAN at risk of rapid disease progression; clinical benefit on kidney function decline has not yet been established and continued approval is contingent on confirmatory evidence [10,62,212].

Sparsentan is a single-molecule dual endothelin-A and angiotensin II type 1 receptor antagonist. Because it provides angiotensin receptor blockade, sparsentan should be considered part of the RAS inhibitor backbone rather than an add-on to it. In the Phase 3 PROTECT trial, sparsentan achieved greater proteinuria reduction than irbesartan and demonstrated preservation of kidney function (eGFR slope) in the confirmatory analysis, supporting conversion to full regulatory approval [62,210]. Real-world data from a 2025 multicenter cohort reported a median 62% UPCR reduction after switching to sparsentan, including in patients already receiving SGLT2 inhibitors [213].

Meta-analyses support class-wide antiproteinuric benefit and slowing of eGFR decline, but efficacy must be balanced against predictable adverse effects. A 2025 systematic review reported a 2.4-fold higher risk of anemia and a signal for fluid retention/edema with ERAs versus placebo [210]. Although hepatotoxicity was more prominent with older agents, contemporary ERAs have generally manageable safety profiles. Overall, ERAs offer an additional non-immunosuppressive option for patients with persistent proteinuria and high-risk features [62].

### 7.4. Combination Therapies and Synergistic Approaches

Combination approaches layer complementary agents on an optimized supportive-care backbone to maximize proteinuria reduction and preserve eGFR [214].

A prominent non-immunosuppressive foundation is integration of hemodynamic therapies, including RAS blockade, SGLT2 inhibitors, and ERAs. In ZENITH-CKD, combined zibotentan plus dapagliflozin produced a 52.5% reduction in urinary albumin-to-creatinine ratio with lower fluid retention than ERA monotherapy, supporting both synergistic efficacy and improved tolerability [215].

Emerging data also support layering disease-modifying immunotherapy onto this nephroprotective backbone. In ORIGIN 3, atacicept reduced proteinuria versus placebo on top of optimized supportive care, with subgroup analyses suggesting consistent efficacy regardless of baseline SGLT2 inhibitor use [197]. Looking ahead, proposed strategies include pairing agents that target distinct steps in the multi-hit cascade (e.g., combining gut-directed therapy with complement inhibition) to address upstream mucosal IgA production and downstream glomerular inflammation [64,216].

Overall, combination therapy in IgAN is shifting toward coordinated, multi-target regimens that integrate hemodynamic protection with pathway-directed immunomodulation in selected high-risk patients [10,214].

### 7.5. Ongoing and Future Clinical Trials in IgAN

**Figure 4:**
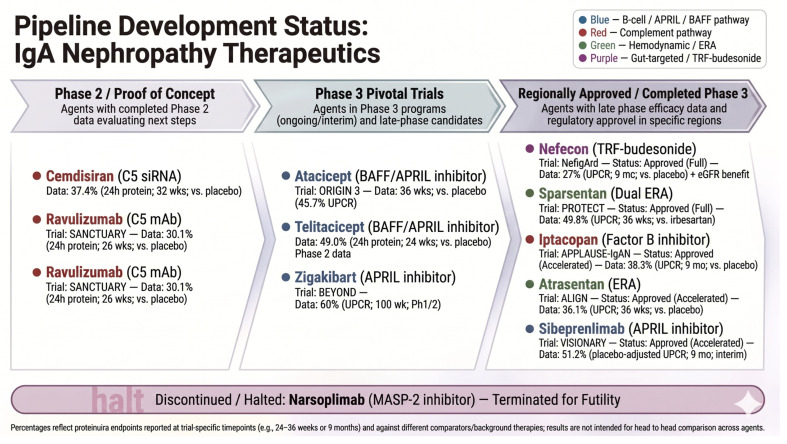
Pipeline development status of IgAN therapeutics. Agents are organized by development stage (Phase 2/proof-of-concept, Phase 3 pivotal programs, and regionally approved/completed Phase 3), with discontinued development shown separately. Colored bullets denote therapeutic class/mechanism: blue = B-cell/APRIL/BAFF pathway; red = complement pathway; green = hemodynamic/endothelin axis (ERAs); purple = targeted-release budesonide. Percent values reflect trial-reported proteinuria endpoints at the stated timepoints and comparators and should not be interpreted as head-to-head comparisons.

Figure 4 summarizes late-phase and regionally approved programs across the BAFF/APRIL axis, complement inhibition, hemodynamic/endothelin modulation, and gut-targeted therapy, and highlights discontinued development. The IgAN pipeline is expanding rapidly, with trials increasingly aligning mechanism-based therapies to the multi-hit model and using standardized endpoints—early proteinuria change (UPCR/24-h protein) with confirmatory eGFR slope—often alongside biomarker enrichment and patient-reported outcomes [200,217]. Beyond the major late-phase programs discussed in Section 7.1, Section 7.2, Section 7.3 and Section 7.4, additional investigational approaches include upstream complement interception (e.g., antisense Factor B suppression in IMAGINATION) and early podocyte-targeted strategies such as transient receptor potential canonical 6 (TRPC6) inhibition [5,217,218].

### 7.6. Pipeline Overview and Unmet Needs

Despite rapid expansion of the IgAN pipeline, major gaps remain. A 2025 landscape synthesis identified 44 active Phase 2/3 adult trials, most using regulatory-aligned surrogate endpoints (UPCR and/or eGFR slope), underscoring increasing standardization across immunologic and non-immunosuppressive programs [219]. However, dedicated antifibrotic strategies remain limited, and further precision in mucosal modulation beyond ileal budesonide is still needed to better suppress Gd-IgA1 generation while minimizing systemic immunosuppression [47,48,220]. Methodologically, progress will depend on better harmonization of surrogate endpoints, incorporation of contemporaneous histologic or molecular profiling at enrollment, and wider use of adaptive multi-arm platforms to test combinations and validate surrogate endpoints efficiently [200,221]. Unresolved questions around sequencing, duration, and class-specific safety monitoring will continue to shape implementation as multi-omics and AI-enabled stratification mature [47,138,217].

## 8. Guidelines and Real-World Implementation

### 8.1. Global Guideline Recommendations

The KDIGO 2025 guidelines serve as the principal global reference for IgAN management, while regional implementation continues to reflect differences in health-system capacity, payer policy, and historical practice patterns [62,222]. In Japan, national guidance continues to support tonsillectomy with steroid pulse therapy in selected patients and is facilitated by longstanding school urinary screening programs [158,223,224]. In China, management broadly aligns with KDIGO but incorporates TESTING-informed lower-dose steroid strategies to balance efficacy against infection risk [154,190]. Across the Middle East and North Africa, constrained access to advanced therapies and workforce limitations often necessitate feasibility-focused pathways centered on supportive care [225]. In the United States, KDOQI commentaries contextualize KDIGO recommendations within payer and coverage complexity, while real-world data suggest persistent underutilization of SGLT2 inhibitors despite guideline endorsement [20,226].

Overall, while global recommendations increasingly converge on shared therapeutic targets, equitable delivery depends on region-specific pathways that address access, affordability, and implementation capacity [222].

### 8.2. Disparities in Implementation

Despite broad consensus on core management principles, implementation of IgAN care remains uneven across regions, largely reflecting differences in diagnostic capacity, access to essential medicines, and policy support, particularly in many low- and middle-income countries (LMICs) [222]. A 2023 systematic review highlighted extreme variation in reported IgAN incidence and attributed much of this contrast to differential biopsy access and ascertainment rather than true absence of disease, creating a form of “data invisibility” in low-resource settings [1]. The high cost of newer agents, including ERAs and complement inhibitors, adds a second layer of inequity: although reimbursement pathways may support uptake in some high-income settings, upfront budget constraints often limit adoption in LMICs even when long-term benefit is plausible [227,228]. System-level policy capacity also varies markedly; the 2025 ISN Global Kidney Health Atlas noted that public funding for dialysis is more common than national early-detection strategies, with only a minority of countries reporting a national strategy for early CKD detection [229,230].

### 8.3. Country-Specific Treatment Patterns

Treatment patterns for IgAN continue to reflect health-system capacity, reimbursement, and clinical traditions despite broadly convergent guidelines (Section 6.1 and Section 7). In Japan, early detection through school urinary screening and continued use of tonsillectomy in selected patients remain distinctive features, whereas China applies KDIGO-aligned care through locally adapted immunosuppression thresholds informed by TESTING [154,158,190,223,224]. Across Africa and parts of South Asia, limited access to diagnostics and essential medicines often constrains care to supportive therapy or symptom-based management while, in Europe, variation more often reflects differences in policy, training, and registry infrastructure [74,222,227,231].

### 8.4. Barriers to Implementation and Adherence

Despite the expanding therapeutic arsenal, major barriers continue to limit equitable delivery of optimal IgAN care, spanning system capacity, financing, and clinical implementation [227].

Economically, the high cost of newer disease-modifying therapies—including targeted-release budesonide, ERAs, and complement inhibitors—restricts access in many LMICs, where public funding is often concentrated on dialysis and transplantation. In high-income countries, payer policies may still require failure of stepwise therapy before authorization of advanced agents, underscoring the need for context-specific policy and reimbursement pathways that preserve access to foundational renoprotective therapy [180,222,229].

Clinically, persistent diagnostic gaps reflect both the invasive nature of kidney biopsy and limited nephrology workforce capacity in many regions, contributing to delayed recognition when fibrosis is advanced and immunomodulation is less likely to be effective. Locally adapted care pathways, task-sharing models, and standardized monitoring may improve deliverability, while validated non-invasive biomarkers may eventually complement biopsy-based diagnosis and monitoring and, if adequately validated, could reduce reliance on invasive assessment in selected settings [1,47,227,230].

Ultimately, achieving proactive, precision-based IgAN care at scale will depend as much on implementation capacity and affordability as on therapeutic innovation [62,64].

### 8.5. Real-World Evidence and Practice Patterns

Real-world evidence (RWE) is increasingly important for complementing randomized trials by characterizing effectiveness, safety, and implementation across broader IgAN populations. Large electronic medical record (EMR)-based analyses highlight the “real-world toxicity” of non-targeted immunosuppression; for example, in a U.S. cohort (n = 4879), systemic glucocorticoid initiation was associated with higher adverse-event rates (including infections and diabetes) and greater healthcare utilization, without a clear kidney survival signal at the population level [232].

Emerging RWE also supports the effectiveness of newer therapies in routine care. Observational studies have reported proteinuria reduction with agents such as finerenone and telitacicept, with signals of eGFR stabilization that are broadly consistent with trial findings, although interpretation remains limited by non-randomized design and short follow-up [233,234].

Robust registry infrastructure strengthens external validity and enables longitudinal outcome benchmarking. The Swedish Renal Registry, which has been validated for biopsy-confirmed IgAN, provides a model for high-quality follow-up, while large registry-based analyses such as CURE-CKD show that system-level factors—including insurance type and prior hospitalization—also shape outcomes [97,231]. In parallel, a 2025 systematic review of 76 clinical studies reinforced the consistency of proteinuria reduction as a surrogate endpoint across drug classes, supporting alignment between routine monitoring and regulatory expectations [139].

Although RWE is limited by missingness and geographic bias, investment in standardized registries remains essential to quantify long-term safety, effectiveness, and cost-effectiveness across the expanding IgAN therapeutic landscape [62].

## 9. Special Populations

Although the core IgAN principles apply broadly, age, pregnancy, and transplantation materially shift risk–benefit trade-offs and monitoring. Children often have less chronic injury at presentation yet remain vulnerable to long-term progression; older adults have greater comorbidity and chronic histopathology that constrain immunosuppression tolerability; pregnancy requires maternal–fetal risk balancing within multidisciplinary care; and post-transplant recurrence is a major contributor to late graft dysfunction. The subsections below summarize population-specific considerations and refer to the diagnostic, risk, and treatment frameworks in Section 4, Section 5, Section 6 and Section 7.

### 9.1. Pediatrics

Across pediatric cohorts (including Oxford/VALIGA groups), segmental sclerosis (S) and tubular atrophy/interstitial fibrosis (T) are the most consistent histologic markers of adverse outcome, while higher time-averaged proteinuria and declining eGFR remain the key clinical indicators of risk despite optimized supportive care [128,235]. Japanese registry data link early and sustained proteinuria remission with superior long-term renal survival, although international cohorts suggest heterogeneity in presentation across ethnic groups [236,237,238]. Current pediatric guidance has shifted away from routine first-line corticosteroids toward a biopsy-informed, risk-adapted approach that prioritizes optimized supportive care, with immunosuppression individualized according to disease severity and safety considerations; historical Japanese trials nevertheless support selective steroid use in severe pediatric disease [86,239,240,241]. Persistent gaps include limited non-Asian pediatric data, transition-of-care challenges, and under-representation of low- and middle-income country cohorts [62,86].

### 9.2. Older Adults

IgAN in older adults is commonly diagnosed against a background of greater comorbidity and more advanced chronic injury, and is associated with poorer renal survival than in younger patients [152,242]. Prognosis is strongly influenced by biopsy features, particularly extensive crescent involvement and vascular/arteriolar lesions coexisting with advanced fibrosis, which are associated with higher risk of kidney failure [243,244,245]. Diagnostic overlap with IgA-dominant infection-related glomerulonephritis is also more common in this age group, making careful clinicopathologic distinction essential [246]. Cohort data indicate lower remission rates, poorer long-term outcomes, and less frequent use of intensive immunosuppression in older adults; accordingly, KDIGO 2025 prioritizes optimized supportive care and comorbidity management, reserving immunosuppression for carefully selected high-risk patients with close safety monitoring [62,85,131,238,247].

### 9.3. Pregnancy

Pregnancy in women with IgAN requires balancing maternal renal health with fetal safety. Outcomes are largely determined by pre-pregnancy kidney function, proteinuria, and blood pressure control; with preserved kidney function and controlled proteinuria, outcomes are generally favorable [248,249]. However, IgAN is associated with increased obstetric risk, particularly preeclampsia and preterm birth, warranting closer surveillance; women requiring antihypertensive therapy before conception appear to be at especially high risk [250,251,252]. KDIGO 2025 emphasizes coordinated nephrology–obstetric care, including discontinuation of teratogenic agents such as RAS inhibitors, SGLT2 inhibitors, ERAs, and MMF before conception, with MMF requiring a 3-month washout and conversion to a pregnancy-compatible alternative when needed [62,253]. Hypertension should be managed with pregnancy-compatible agents, low-dose aspirin is recommended for preeclampsia prophylaxis and, if immunosuppression is required, acceptable options include low-dose corticosteroids, azathioprine, and calcineurin inhibitors. Safety data for newer IgAN therapies during pregnancy and lactation remain limited, so medication planning should extend into the postpartum period [62,253]. Overall, pregnancy is feasible for many women with well-controlled IgAN when medication adjustments and multidisciplinary follow-up are established before conception [62].

### 9.4. Recurrent IgAN Post-Transplant

Recurrence of IgAN after kidney transplantation is common on protocol biopsy (≈20–50% in contemporary series), whereas clinically significant recurrence that affects graft outcomes is less frequent; distinguishing histologic recurrence from clinically meaningful disease is therefore central to post-transplant management [134,254,255,256]. Risk is higher in younger recipients, living-related donor transplants, and those with aggressive native kidney disease or rapid progression to kidney failure, although recurrence remains heterogeneous and many cases follow an indolent course [131,133,136,256,257]. Preventive strategies remain controversial, with conflicting registry data on steroid maintenance and no proven prophylactic approach [257,258]. In practice, tacrolimus-based regimens remain the calcineurin inhibitor backbone, and optimized supportive care—particularly RAS blockade—has been associated with lower proteinuria and improved graft outcomes in recurrent disease [129,134,259]. Long-term cohort data indicate that recurrence contributes meaningfully to late graft loss, often beyond 10 years post-transplant, especially in younger recipients; accordingly, KDIGO 2025 prioritizes supportive care and comorbidity optimization, reserving immunosuppression modification for carefully selected patients given the heightened infectious and metabolic risks in transplant recipients [62,260].

## 10. Gaps in Knowledge

### 10.1. Limitations in Risk Stratification Models and Histopathology

Risk prediction tools in IgAN remain valuable for prognostication and trial enrichment, but they should not be used as stand-alone determinants of treatment decisions; the main tools and their clinical application are summarized in Section 5.

A major limitation is histologic reproducibility. Inter-observer variability within the Oxford MEST-C classification persists, with the weakest agreement reported for endocapillary hypercellularity and crescents and only moderate concordance for mesangial hypercellularity, segmental glomerulosclerosis, and tubulointerstitial fibrosis, limiting cross-center comparability and the consistency of histology-anchored risk scoring [114,261].

A second gap is calibration drift and population-dependent performance. As treatment standards evolve, models derived in earlier eras may misestimate risk in contemporary cohorts; recent validations suggest overestimation in patients treated with newer agents and potential miscalibration at longer time horizons or at the extremes of baseline risk, supporting periodic recalibration across settings [95,262,263].

Consistent with these constraints, KDIGO 2025 emphasizes that histology-based scores and risk models should inform—but not dictate—individualized treatment decisions [62].

Priorities therefore include improving histology reproducibility (digital pathology standard operating procedures and AI-assisted scoring), recalibrating models in contemporary therapy eras and under-represented populations, and prospectively validating integrative models that combine histology with biomarkers before broad adoption in routine care or trial stratification [114].

### 10.2. Therapeutic Evidence Gaps

Despite an expanding pipeline, pivotal IgAN trials still rely predominantly on surrogate endpoints for regulatory approval (see Section 7.6), limiting direct evidence for effects on kidney failure and mortality. KDIGO 2025 notes that while such surrogates can support regulatory approval, they may not fully establish long-term efficacy on hard outcomes [9,62].

Long-term durability and safety data remain a central gap. Targeted-release budesonide (NefIgArd) and sparsentan (PROTECT) have demonstrated sustained proteinuria reductions with favorable short-term eGFR slope signals, but evidence beyond approximately 2–3 years is still maturing [163,264]. In parallel, evidence for SGLT2 inhibitors in IgAN is largely extrapolated from broader CKD trials; although exploratory strata (e.g., in ALIGN) suggest additivity with endothelin blockade, these analyses remain underpowered for definitive inference [10].

Additional gaps reflect both trial design and enrolled populations. Direct head-to-head comparisons between novel classes (e.g., ERAs vs. B-cell modulation) are lacking, and pivotal programs commonly exclude children, patients with advanced CKD (eGFR < 30 mL/min/1.73 m^2^), and transplant recipients—limiting generalizability to populations with high unmet need. Finally, although combination approaches are increasingly used in practice, rigorous trials defining optimal sequencing and pairing strategies remain limited.

Future research priorities therefore include pragmatic, longer-duration trials that incorporate composite hard endpoints (kidney failure, death) alongside patient-reported outcomes. Platform or factorial designs could accelerate testing of combinations and sequences, while intentional inclusion of pediatric and advanced CKD populations will be important to improve generalizability across clinically relevant subgroups [10,163].

### 10.3. Special Populations and Transplantation

Evidence for pediatrics, older adults, advanced CKD, and transplant recipients with IgAN remains limited. While clinical features and management nuances are detailed in Section 9.1, Section 9.2, Section 9.3 and Section 9.4, key evidence gaps persist.

Pediatrics: Children are consistently under-represented in trials despite presenting with more active and fewer chronic lesions at diagnosis. Consequently, much of their management evidence is extrapolated from adults. The IPNA 2024 guidelines endorse supportive care as the first line, recommending cautious, individualized steroid use given the low certainty of evidence for aggressive immunosuppression [86,265].

Older Adults: Older adults face higher mortality rates and a variable burden of chronic histologic lesions. However, they often receive less intensive immunosuppression due to concerns regarding comorbidity and frailty, a practice pattern supported by observational data showing increased toxicity risks [242,246,247].

Advanced CKD: Patients with advanced CKD (eGFR < 30 mL/min/1.73 m^2^) are routinely excluded from pivotal trials (e.g., NefIgArd, PROTECT), leaving uncertain benefit from proteinuria-lowering strategies and a narrow role for immunosuppression. Management in this group should be highly individualized, with priority given to supportive measures to delay dialysis [62,163,264].

Transplantation: After transplantation, IgAN recurrence is common and variably pathogenic, with no established or uniformly effective prophylaxis. Recurrence significantly increases the risk of graft failure and is more likely in younger recipients, living-related donors, and those with aggressive native disease [136,257].

Collectively, KDIGO 2025 urges prioritization of these groups in future research, along with broader inclusion from LMICs to improve generalizability. Research priorities include dedicated age/stage-specific trials with standardized outcomes, prophylaxis studies for post-transplant recurrence, and biomarker-guided selection to improve external validity and health equity [62,216].

## 11. Future Research Directions

Based on the evidence reviewed in Section 5, Section 6, Section 7, Section 8, Section 9 and Section 10, the priorities below summarize the key opportunities to strengthen risk prediction, align therapy selection with disease biology, and improve real-world implementation.

### 11.1. Refining Risk Stratification and Biomarkers

Priorities include prospective validation of biomarkers and multi-omic signatures across diverse cohorts, harmonization of biospecimen collection and data standards to enable cross-cohort comparability, and integration of reproducible digital pathology and machine learning tools to reduce variability in histologic assessment and support individualized risk prediction.

### 11.2. Optimizing Therapeutic Strategies

Key needs include pragmatic, longer-duration trials that reflect contemporary background therapy; head-to-head comparisons where feasible; and studies that define optimal sequencing, combination, and duration of emerging agents. Trials should pair kidney-relevant outcomes with PROs capturing symptom burden, mental health, and life participation, while ensuring deliberate inclusion of under-represented groups and clinically relevant subpopulations.

### 11.3. Enhancing Global and Equitable Representation

Multi-region programs should broaden enrollment beyond current geographic concentrations, harmonize biopsy practice and outcome assessment, and embed implementation research to address access, affordability, and monitoring constraints across health systems.

### 11.4. Methodologic Innovation for Efficient Evidence Generation

Priorities include standardized biobanking and data sharing to enable reproducible omics and machine learning research, prospective external validation of predictive models, and clearer pathways for clinical implementation; adaptive or platform trial designs may further accelerate evaluation of therapies and combinations.

## 12. Conclusions

IgAN is a heterogeneous disease with highly variable progression, making risk-aligned care essential to identify patients who warrant early treatment escalation while avoiding unnecessary toxicity in those at lower risk. This review frames prognosis as a longitudinal construct that integrates proteinuria and eGFR trajectories with histopathology and risk tools, while acknowledging inter-observer variability within Oxford MEST-C and calibration drift in prediction models in the context of contemporary therapy. Within this framework, optimized supportive care is the foundation and platform for subsequent therapies. This includes intensive blood pressure control with maximal tolerated ACEi/ARB, dietary sodium restriction and lifestyle measures, and SGLT2 inhibitors for eligible patients. Immunomodulatory approaches are positioned as selective and safety-conscious: systemic corticosteroids remain an option for carefully chosen higher-risk patients with persistent proteinuria despite optimized supportive measures, but potential benefit must be balanced against metabolic complications and infection risk, supporting reduced-dose strategies, antimicrobial prophylaxis, and structured monitoring. Targeted-release budesonide (Nefecon) is presented as a targeted option when limiting systemic glucocorticoid exposure is a priority, alongside explicit caution against assuming interchangeability with other oral budesonide formulations, while non-steroidal immunosuppressants have narrower, context-dependent roles and require thoughtful risk mitigation.

The expanding pipeline reflects a shift toward mechanism-aligned treatment across key nodes of the multi-hit model, spanning BAFF/APRIL axis targeting, complement pathway therapies across alternative, lectin, and terminal/central components, and hemodynamic/endothelin modulation, with growing interest in sequencing and combination strategies on top of supportive care, although optimal sequencing remains undefined. Trials increasingly use standardized endpoints of early proteinuria change with confirmatory eGFR slope, often alongside biomarker enrichment and patient-reported outcomes, yet pivotal evidence remains heavily dependent on surrogate endpoints and evidence beyond approximately 2–3 years is still maturing for several major programs. The priorities highlighted in this manuscript include pragmatic longer-duration studies with hard endpoints, clearer evidence to guide sequencing and combinations, improved non-invasive biomarkers that may complement biopsy-based diagnosis and monitoring and, if adequately validated, could reduce reliance on invasive assessment in selected settings, and deliberate inclusion of under-represented populations and clinically relevant subgroups, including advanced CKD and transplantation. Finally, translation into routine practice will be constrained by diagnostic capacity, affordability, and regional access disparities; strengthening real-world evidence through registries and implementation efforts is therefore integral to ensuring that therapeutic innovation yields equitable benefit. The take-home message is to anchor care in optimized supportive therapy, deploy immunomodulation judiciously with structured toxicity risk mitigation, and build durable, globally generalizable evidence that aligns mechanism-based treatments with the right patients and outcomes that matter.

## Figures and Tables

**Table 3:** Risk Prediction Models in IgAN (Adults and Pediatrics).

Model/Study (Year)	Cohort and Population	Inputs/Predictors	Output/Predicted Outcome	Strengths	Limitations/Notes
International IgAN Prediction Tool (IIgAN-PT) [90,91]	Multinational adult cohorts (Europe, Asia, North America); validation in >4000 patients	Age, sex, race, eGFR, proteinuria, MAP, MEST-C score, baseline immunosuppression	5-year risk of ≥50% eGFR decline or ESKD	Global standard; web calculator available; validated in long-term (12-year) cohorts	Overestimates risk in contemporary cohorts treated with SGLT2 inhibitors or sparsentan; inclusion of race as a variable remains debated.
Oxford MEST-C histology [65,73])	International biopsy cohorts; recent validation in Hispanic populations	M, E, S, T, C	Progression risk (adverse renal outcomes)	Standardized reporting; S and T are strongest independent predictors across ethnicities	Not a standalone calculator; C1 lesions may be treatment-responsive, while C2 predicts poor outcomes.
Pediatric prediction models [111,130]	Pediatric cohorts (Korea, North America, Europe)	2-year proteinuria trajectory; recalibrated IIgAN-PT variables for children	Kidney survival; risk of function loss	Trajectory outperforms baseline proteinuria; recalibration improves discrimination in Asian children	Standard adult IIgAN-PT may miscalibrate risk in children without age-specific adjustment.
Primary care risk model [106]	Japanese multicenter cohort (*n* = 1174)	Clinical only: eGFR, proteinuria, hematuria, age, sex (no biopsy)	Probability of ESKD	Enables stratification where biopsy is not feasible; C-statistic ~0.80	Slightly lower accuracy than histology-based models; primarily validated in Japanese cohorts
AI and multi-omics models [108,124]	Retrospective cohorts; training + testing sets	Longitudinal inputs (IMV-LSTM); network biomarkers (KMN) + metabolomics	Dynamic risk trajectories; cluster-based risk	Higher accuracy (AUC 0.77–0.93) than standard tools; captures dynamic change	Requires complex inputs (omics/serial data); limited clinical implementation to date
XGBoost prognostic nomogram [101]	Retrospective IgAN cohorts; training + internal validation with independent external validation cohort	eGFR stage, 24 h proteinuria, Oxford T score, hypertension, anemia	5-year renal survival/progression risk (per study definition)	Limited set of routinely available predictors; nomogram format supports usability; includes independent external validation	Retrospective design; risk of overfitting (low event-per-variable); endpoint definition and treatment-era differences may limit comparability with IIgAN-PT; requires prospective validation in contemporary therapy cohorts

Abbreviations: MAP = mean arterial pressure.

## Data Availability

No new data were created or analyzed in this study. Data sharing is not applicable to this article.
